# A novel method for detecting brown planthopper (*Nilaparvata lugens* Stål) early infestation using dual-temporal hyperspectral images

**DOI:** 10.3389/fpls.2025.1680474

**Published:** 2025-10-31

**Authors:** Xuying Huang, Shun Jiang, Shanshan Feng, Lei Zhang, Yangying Gan, Lianlian Hou, Chengrui Mao, Ruiqing Chen, Hanxiang Xiao, Yanfang Li, Zhanghua Xu, Canfang Zhou

**Affiliations:** ^1^ Institute of Agricultural Economics and Information, Guangdong Academy of Agricultural Sciences, Guangzhou, China; ^2^ Key Laboratory of Urban Agriculture in South China, Ministry of Agriculture and Rural Affairs, Guangzhou, China; ^3^ Plant Protection Research Institute, Guangdong Academy of Agricultural Sciences, Guangzhou, China; ^4^ Academy of Geography and Ecological Environment, Fuzhou University, Fuzhou, China

**Keywords:** brown planthopper, rice, paddy, remote sensing, hyperspectral, UAV

## Abstract

Accurate and prompt monitoring of brown planthopper (BPH) infestation is crucial for rice production stability. The unique advantages of remote sensing in mapping the location and severity of pest damage are widely acknowledged. However, the crypticity of BPH early damage complicates the identification of infested areas. This study aims to detect BPH early infestation in paddy fields using an unmanned aerial vehicle (UAV) hyperspectral imaging system. Two data acquisition campaigns were conducted during the BPH early infestation stage. Considering the dynamic spatial distribution of BPH, the pest population density records were averaged to indicate infestation severity during the investigation period. Three novel indices were designed to detect the BPH early damage. Specifically, the Dual-temporal Stressed Canopy Spectral Relative Difference Index (DSRI) and the Dual-temporal Stressed Canopy Spectral Direct Difference Index (DSDI) were proposed based on the dual-temporal spectral changes of rice canopy. Furthermore, an opposite trend of DSDI in the short-wavelength (399–750 nm) and long-wavelength (750–1006 nm) spectral regions was observed for samples with varying BPH severity. Thus, the DSDI-SL was further proposed. The optimal feature combination of DSRIs, DSDIs and DSDI-SLs was selected using Lasso regularization and recursive feature elimination (RFE). An XGBoost classifier was applied to establish the BPH early detection model, which achieved an overall accuracy (OA) of over 85%, outperforming the model established by mono-temporal collected data. In the context of global climate change and escalating challenges to food security, our research introduces a novel framework for the efficient detection and quantitative description of early-stage BPH damage.

## 1 Introduction

Rice accounts for 26.2% of the global yield of grain and oil crops, serving as the primary dietary staple for over half of the global population. China, as the world’s largest producer of rice, contributes approximately 30% to the global annual rice production (Fao, 2024). The brown planthopper (*Nilaparvata lugens* Stål, BPH) is one of the most destructive pests to rice, infesting nearly 26.6 × 10^4^ km^2^ of paddy fields annually in China (Guo et al., 2023). BPH infestation substantially reduces grain weight, causing yield losses of 10%-80% and even total crop failure (Liu and Sun, 2016; Balachiranjeevi et al., 2019; Satturu et al., 2020; Jeevanandham et al., 2023). Therefore, there is an urgent need to establish a robust monitoring system for BPH infestation to facilitate early warning and control efforts.

The BPH is a typical phloem-feeding insect, making it highly cryptic as its feeding sites are normally located on the stems of rice plants (Sriram et al., 2024). When visible degeneration of the rice phenotype occurs, it usually signifies that the BPH population density has already surpassed the warning threshold for pest control (Yu et al., 2022; Choi et al., 2025). Accurately and promptly locating BPH occurrences and assessing their severity are fundamental for implementing targeted control measures. Currently, the assessment of BPH damage still primarily relies on manual field surveys. This approach is time-consuming and labor-intensive, permitting only the collection of sampling statistics from small areas. Pest forecasting lamps can provide information on the timing of outbreaks and the relative population density of BPH, but fails to delineate the precise locations and severity of infestations (Zhang and Cheng, 2013; Wan et al., 2016). In contrast, remote sensing provides spatiotemporally continuous observations of paddy fields, showing great potential for detecting BPH infestations (Bai et al., 2023; Xia et al., 2024; Yuan et al., 2025; Zhang et al., 2025).

Generally, imaging data from aerial or satellite remote sensing platforms are unable to directly capture insect information. Instead, the spatial distribution and pest severity are indirectly detected through phenotypic changes in the host plants (Zhang et al., 2019). BPH pierces the phloem of rice plants with its stylet (a needle-like mouthpart) to suck sap, leaving a hollow stylet sheath *in situ* after feeding, which obstructs nutrient transport within the rice plants (Zhao et al., 2023; Hu et al., 2024). The deficiency of water and inorganic salts hampers the ability of rice plants to synthesize photosynthesis-required pigments, leading to symptoms such as yellowing and wilting, and increasing the risk of secondary disasters (Zheng et al., 2023). Most current research predominantly focuses on the mechanisms of resistance in rice involving endogenous hormones and genes under BPH infestation (Lu et al., 2022; Chen et al., 2023; Xu et al., 2024). Findings regarding the use of rice canopy phenotypic traits to detect the spatial distribution of BPH are limited. Several studies have investigated the detectability of BPH damage using remote sensing techniques through controlled experiments. For instance (Xiong et al., 2024), demonstrated that near-infrared (NIR) reflectance of the rice canopy, temperature differences between the canopy and air, and leaf chlorophyll content are significantly negatively correlated with BPH population density. (Tan et al., 2019) established that the ratio index derived from the red-edge spectral region can serve as a reliable indicator for both the physiological compensation and the subsequent stress responses of rice plants to BPH infestation. However, the complex paddy habitat, coupled with the crypticity of BPH damage, complicates the direct application of these findings to field conditions (Zhang et al., 2023; Mochizuki et al., 2024).

A few studies have endeavored to detect BPH damage utilizing medium-resolution satellite optical imagery, e.g., Landsat, SPOT (Ghobadifar et al., 2016a, b). Nonetheless, the challenges posed by the long revisit cycles of satellites and frequent cloud cover limit the usability of optical satellite data for monitoring BPH damage during critical periods. Additionally, the relatively coarse spatial and spectral resolutions may dilute the signals from host plants, potentially leading to the omission of crucial features associated with rice plants under mild BPH stress, e.g., low pest densities, the early stages of infestation (Chen et al., 2024). Unmanned aerial vehicle (UAV) enhance flexibility in data acquisition timing. Equipped with high-throughput imaging sensors, UAV-based data are capable of delivering detailed phenotypic information on host plants during critical pest infestation stages, thereby offering valuable insights for pest localization and damage severity assessment (Hongo et al., 2024; Xia et al., 2024). Yet, to our knowledge, no prior studies have utilized UAV data specifically for detecting BPH early damage.

Since BPH does not directly attack the leaves, the phenotypic traits of the rice canopy change marginally during the early stages of BPH infestation (Yang et al., 2024). It has been revealed that the physiological characteristics of rice exhibit delayed response to BPH damage (Chen and Liu, 2023). Therefore, it is essential to capture the optical signals of rice plants under mild stress conditions. Otherwise, the practical applicability of monitoring results could be notably diminished. Another challenge arises from the dynamic spatial distribution of BPH populations in field conditions. In controlled experiments, BPH density within each rice cluster is typically consistent. In contrast, BPH populations exhibit considerable spatiotemporal variability in paddy field due to various driving factors, e.g., habitat diversity, reproductive dynamics (Rashid et al., 2016, 2017). This implies that the phenotypic traits of infested rice canopies might not accurately correlate with the BPH damaged severity recorded concurrently. Consequently, the impact of the dynamic changes in pest population densities should be considered when remotely detecting BPH damage.

Although previous studies have explored the detectability of remote sensing for monitoring BPH infestation at different scales, its effectiveness in identifying this pest at early stages under field conditions remains uncertain. To address this issue, we propose a novel method for detecting BPH during the early infestation period using dual-temporal hyperspectral UAV images. Specifically, we (1) assess the uncertainty of remotely detected results of BPH infestation using mono-temporal spectral-based features; (2) propose a novel method for detecting BPH infestation based on dual-temporal spectral differences of the rice canopy; (3) evaluate the applicability of the proposed method in identifying varying degrees of BPH severity at the early stage of infestation.

## 2 Materials and methods

### 2.1 Study area

**Figure 1:**
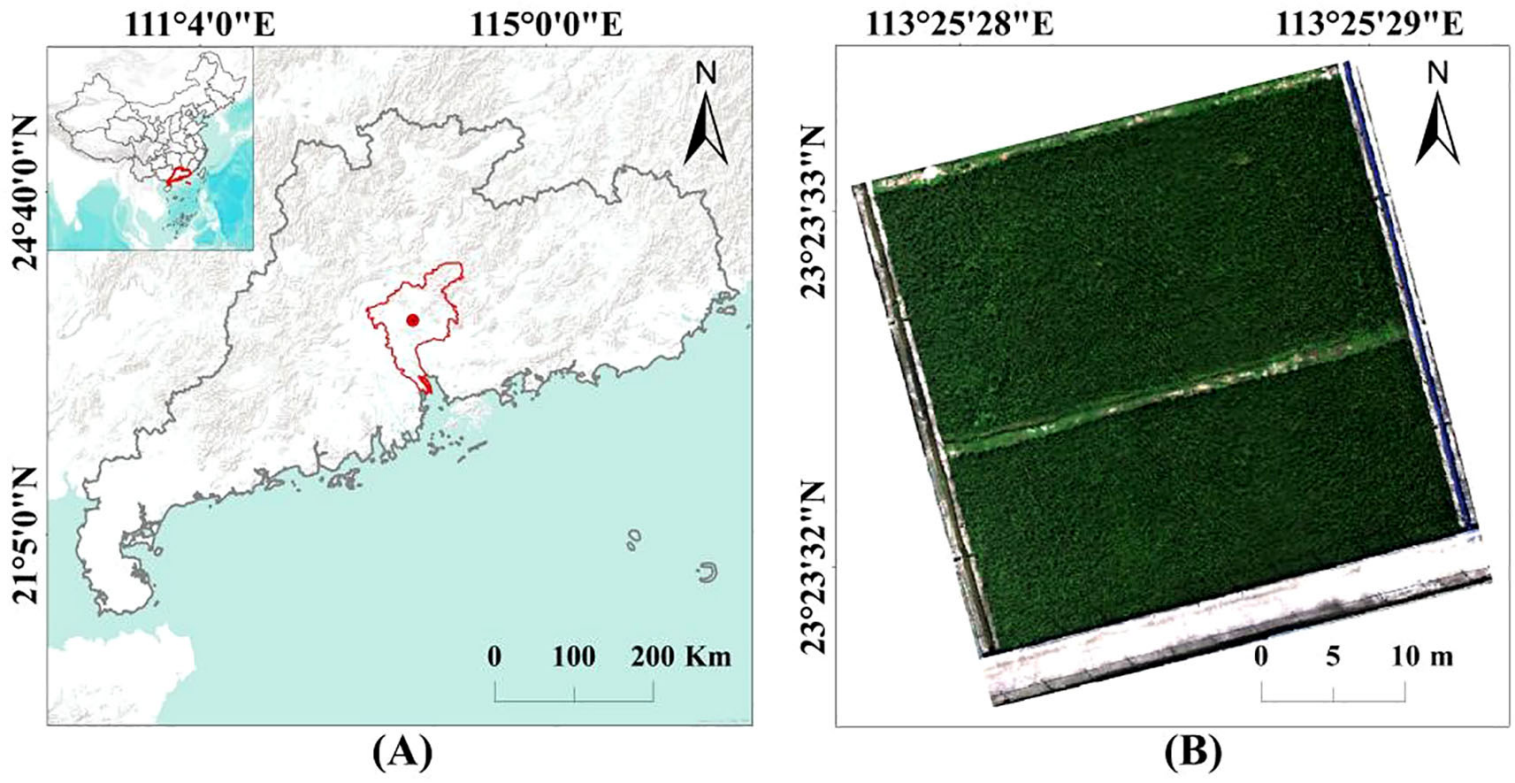
**(A)** Experimental location and **(B)** research paddy field.

The research paddy field is located at the Baiyun Experimental Base, Guangdong Academy of Agricultural Sciences (23°39’ N, 113°42’ E, **Figure 1**). The area has a warm and humid climate, with an annual average temperature of about 23°C, relative humidity of approximately 74.8%, and about 1906 hours of sunshine annually. The average annual precipitation is approximately 1700 mm. The research paddy field spans an area of approximately 1135 m², and the rice variety used in the experiment is ‘Nanjing Xiang Zhan’.

### 2.2 Data collection and processing

#### 2.2.1 Field investigation

**Figure 2:**
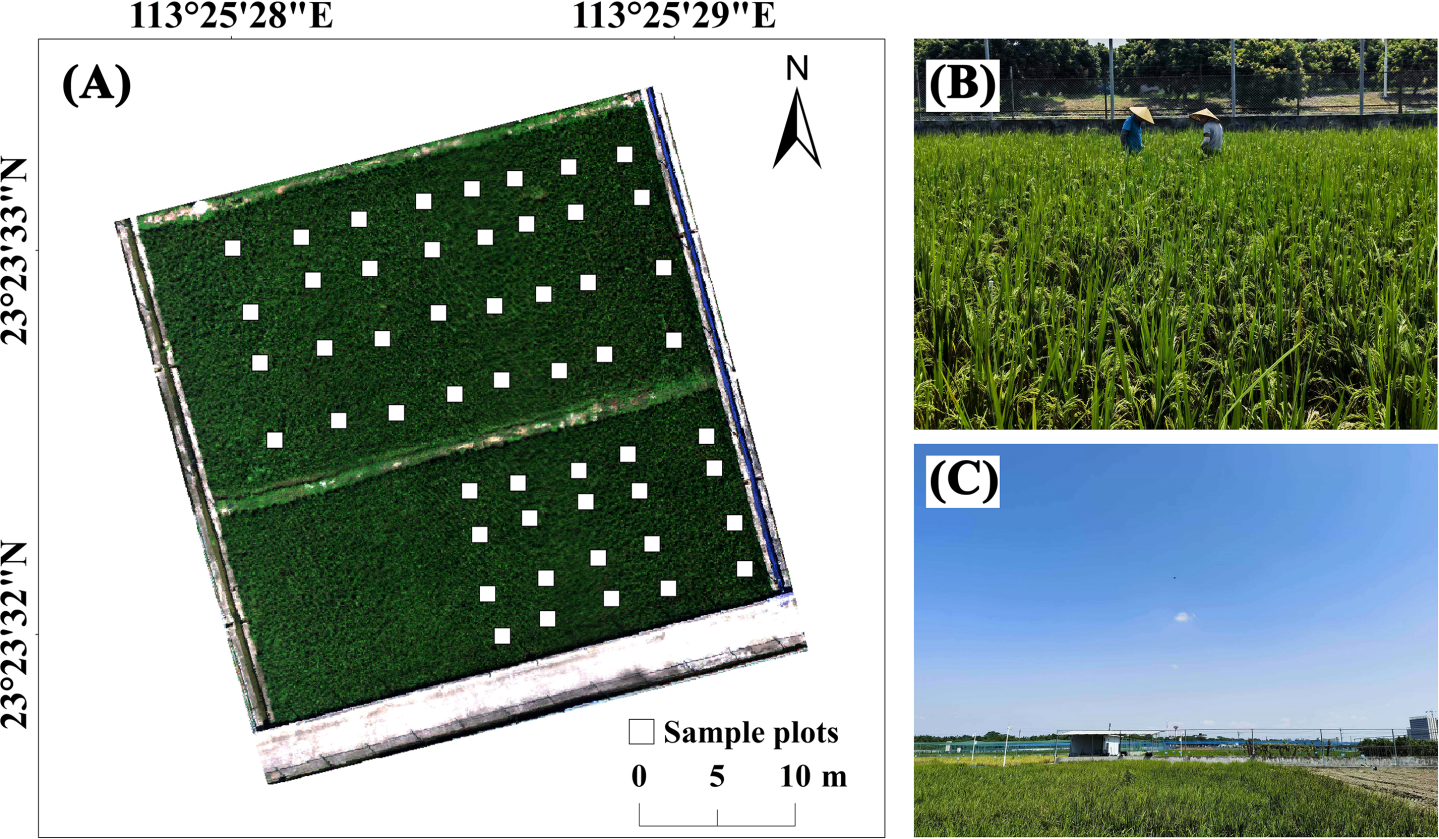
**(A)** Location of the plots within the paddy field. The southwest corner was reserved for other experiments, and no observation plots were established there. **(B)** Rice morphology during field survey (no visible symptoms in damaged rice). **(C)** Weather conditions during UAV hyperspectral image acquisition.

The observational experiment was conducted during the late rice planting season on September 30 (T1) and October 8 (T2), 2024, when the rice was at the heading stage. To minimize interference from other pests and diseases, targeted control measures were consistently implemented within the research paddy field. A total of 52 observation plots, each measuring 1m × 1m, were distributed throughout the field (**Figure 2**). Based on alerts from the pest forecasting lamp at the experimental base, the investigation was conducted during the early stage of BPH infestation. No visible phenotypic degradation of the rice was observed during this period.

Five clumps of rice located at the four corner points and the center of each observation plot were selected as sampling targets. The number of BPH in each clump was counted, and the average was calculated to represent the BPH population in the plot. Comprehensively referencing the “Rules of investigation and forecast for the rice planthopper (*Nilaparvata lugens* Stål and *Sogatalla furcifera* Horváth) (GB/T 15794-2009)” and the “Technical regulations for comprehensive control of major pests affecting high quality rice in Guangdong (DB44/T 2212-2019)”, the averaged BPH population was classified into three levels: mild infestation (< 5 individuals per clump), moderate infestation (5–10 individuals per clump), and severe infestation (> 10 individuals per clump).

#### 2.2.2 UAV hyperspectral images collection and preprocessing

The UAV hyperspectral data collection was conducted simultaneously with the ground-based field survey. Flight times ranged from 11:00 AM to 2:00 PM under sunny conditions. A 300 TC hyperspectral camera (Yiruisi Remote Sensing Technology Co., Ltd., Beijing, China) was mounted on a DJI M300 quadcopter platform (DJI Technology Co., Ltd., Shenzhen, China) to capture hyperspectral imagery of the paddy field. The 300 TC camera comprises 304 bands, providing spectral information ranging from 399–1006 nm, with a spectral resolution of 2 nm. The UAV flew at an altitude of 50 m above ground level, producing an image spatial resolution of approximately 5 cm. The lateral and heading spatial overlaps of 70%. A standard whiteboard was placed at the edge of the paddy field during the flight for reflectance calibration. Additionally, a UAV multispectral orthophoto was captured as reference data for geographic registration and localization of the observation plots.

The hyperspectral raw data were radiometrically calibrated using the calibration files provided by MegaCube 2.14.0 (Yiruisi Remote Sensing Technology Co., Ltd., Beijing, China). Based on the multispectral reference orthophoto, the hyperspectral images were registered and mosaicked in ArcMap 10.4 and ENVI 5.3.1. The mosaicked images were then imported into MegaCube software to generate a hyperspectral hypercube. Finally, the digital number (DN) values of the hyperspectral images were converted to reflectance using the ground-based standard whiteboard.

Based on the positions of the observation plots provided by the orthophoto, the vector files (1m × 1m square, 441 pixels) of each plot were delineated on the hyperspectral images. Pixels within these vector files were extracted as analysis samples, with their labels corresponding to the BPH infestation severity of each plot.

### 2.3 Mono-temporal spectral-based BPH identification features

Since BPH infestation can induce physiological changes in rice, 24 vegetation indices (VIs) associated with vegetation biochemical components and structural characteristic were selected to assess the effectiveness of mono-temporal spectral-based features in detecting BPH infestation severity recorded concurrently (**Table 1**).

**Table 1:** Details of the vegetation indices used for BPH monitoring in mono-temporal scenario.

Vegetation indices abbr.	Formula	References
Photochemical Reflectance Index, PRI	$\left(R_{531}-R_{570})/(R_{531}+R_{570}\right)$	(Penuelas et al., 1994)
Normalized Green-Red Difference Index, NGRDI	$(R_{550}-R_{665})/(R_{550}+R_{665})\;$	(Tucker, 1979)
Green Leaf Index, GLI	$\frac{\left(R_{550}-R_{665})+(R_{550}-R_{490}\right)}{\left(R_{550}+R_{665})+(R_{550}+R_{490}\right)}$	(Louhaichi et al., 2001)
Chlorophyll Index Green, CIG	$R_{865}/R_{550}-1$	(Gitelson et al., 2003)
Normalized Difference Vegetation Index, NDVI	$\left(R_{865}-R_{680})/(R_{865}+R_{680}\right)$	(Rouse et al., 1973)
Chlorophyll Vegetation Index, CVI	$\left(R_{865}\times R_{705})/(R_{550}\times R_{550}\right)$	(Vincini et al., 2008)
Anthocyanin Reflectance Index, ARI1	$1/R_{550}-1/R_{700}$	(Gitelson et al., 2001)
Anthocyanin Reflectance Index, ARI2	$R_{800}\times (1/R_{550}-1/R_{700})$	(Gitelson et al., 2001)
Green Normalized Difference Vegetation, GNDVI	$\left(R_{780}-R_{550})/(R_{780}+R_{550}\right)$	(Gitelson and Merzlyak, 1998)
Normalized Difference Red Edge Index, NDRE	$\left(R_{790}-R_{720})/(R_{790}+R_{720}\right)$	(Cao et al., 2019)
Red Edge Inflection Point, REIP	$\frac{700+40\times [0.5\times (R_{720}+R_{720})-700]}{R_{740}-R_{700}}$	(Curran et al., 1995)
Modified Chlorophyll Absorption Ratio Index, MCARI	$\frac{[R_{700}-R_{670}-0.2\times (R_{700}-R_{550})]\times R_{700}}{R_{670}}$	(Daughtry et al., 2000)
Modified Red Edge Normalized Difference Vegetation Index, MRENDVI	$\left(R_{750}-R_{705})/(R_{750}+R_{705}-2\times R_{445}\right)$	(Sims and Gamon, 2002)
Modified Red Edge Simple Ratio, MRESR	$\left(R_{750}-R_{445})/(R_{705}-R_{445}\right)$	(Sims and Gamon, 2002)
Modified Triangular Vegetation Index, MTVI	$1.2\times [1.2\times (R_{800}-R_{550})-2.5\times (R_{670}-R_{550})]$	(Haboudane et al., 2004)
Triangular Vegetation Index, TVI	$60\times (R_{750}-R_{550})-100\times (R_{670}-R_{550})$	(Broge and Leblanc, 2001)
Vogelmann Red Edge Index 1, VERI1	$R_{740}/R_{720}$	(Vogelmann et al., 1993)
Vogelmann Red Edge Index 2, VERI2	$\left(R_{734}-R_{747})/(R_{715}+R_{726}\right)$	(Vogelmann et al., 1993)
Structure Insensitive Pigment Index, SIPI	$\left(R_{800}-R_{445})/(R_{800}-R_{680}\right)$	(Penuelas et al., 1995)
Plant Senescence Reflectance Index, PSRI	$(R_{680}-R_{500})/R_{750}$	(Merzlyak et al., 1999)
Carotenoid Reflectance Index 1, CRI1	$1/R_{510}-1/R_{550}$	(Gitelson et al., 2002)
Carotenoid Reflectance Index 2, CRI2	$1/R_{510}-1/R_{700}$	(Gitelson et al., 2002)
Chlorophyll Sensitive Index, CSI	$2.5\times \frac{R_{490}}{R_{705}}\times \frac{R_{865}-R_{705}}{R_{865}+R_{705}}$	(Zhang et al., 2022)
LAI-insensitive Chlorophyll Index, LICI	$\frac{R_{735}}{R_{720}}-\frac{R_{573}-R_{680}}{R_{573}+R_{680}}$	(Li et al., 2020)

### 2.4 Dual-temporal spectral difference-based BPH identification features

#### 2.4.1 Construction of dual-temporal spectral difference-based indices

Considering the spatial dynamic variability of BPH distribution, the pest population counts from two separate investigation dates were averaged to denote the infestation degree during this period. These averaged counts were then classified into three levels based on the criteria for BPH infestation severity outlined in section 2.2.1.

The development of the indices was guided by two assumptions: (1) the spectral changes of healthy rice during growth follow predictable trends, whereas BPH infestation alters the magnitude of such changes; (2) if rice is damaged by BPH (i.e., its nutrient transport system is impaired), its spectral variation patterns are expected to differ from those of healthy rice. For example, suppose the NIR reflectance of a rice clump is 0.25 at T1. Under healthy growth conditions (before maturity), it would normally increase to 0.30 at T2 (Prabhakar et al., 2024). However, whether a BPH infestation occurred during the subsequent growth period or was already present before T1, the NIR reflectance of damaged rice at T2 may increase only to 0.28 or even decrease. Thus, the spectral differences between T1 and T2 provide a basis for detecting BPH infestation.

To comprehensively characterize the reflectance changes in the rice canopy caused by BPH infestation, the Dual-temporal Stressed Canopy Spectral Relative Difference Index (DSRI) and the Dual-temporal Stressed Canopy Spectral Direct Difference Index (DSDI) were proposed. DSRI emphasizes the relative spectral changes of the damaged rice canopy, which can reduce the impact of varying environmental illumination on detection results. In contrast, DSDI emphasizes the magnitude of direct spectral changes in damaged rice canopy, allowing sensitive detection of BPH-induced spectral variations. When calculating DSDI, it is important to note that the reflectance of shaded and sunlit leaves differs considerably. Directly using the dual-temporal reflectance differences as indicators for BPH identification can introduce substantial uncertainty. To mitigate this effect, each pixel’s difference spectrum is normalized to the range [0, 1] using min-max normalization. The formulas for calculating DSRI and DSDI are as follows (Equations 1, 2):


(1) $$\text{DSRI}\;=\;(R_{late}-R_{early})/(R_{late}+R_{early})$$

(2) $$\text{DSDI}\;=\;Norm_{\min-\max}(R_{late}-R_{early})$$

where *R_early_* and *R_late_* denote the reflectance of rice canopy collected at T1 and T2, respectively. *Norm_min-max_* denotes the min-max normalization. Calculation examples of these two indices are provided as follows. Assuming that the rice canopy reflectance at 800 nm is 0.25 at T1 and 0.30 at T2, DSRI for this band is calculated as (0.3 - 0.25)/(0.3 + 0.25) = 0.09. For DSDI, the reflectance difference between T1 and T2 is first calculated (e.g., 0.3 - 0.25 = 0.05), and then a min-max normalization is performed on the difference spectrum of each pixel.

Distinctly opposite trends in the DSDI were observed across different BPH infestation severity in the short-wavelength (399–750 nm) and long-wavelength (750–1006 nm) spectral regions (refer to Section 3.1 for details). Based on these observations, the DSDI-SL was further developed. The construction process is as follows:

1. Linear Discriminant Analysis (LDA), SHapley Additive Explanations (SHAP), and Analysis of Variance (ANOVA) were collectively employed to select DSDI from representative bands as candidate factors for the construction of DSDI-SL. LDA selects the most discriminative factors for BPH severity identification by maximizing the ratio of between-class variance to within-class variance (Fisher, 1936). SHAP provides feature importance explanations by quantifying the contribution of each factor to model predictions, which is particularly useful for handling nonlinear relationships and high-dimensional data (Lundberg and Lee, 2017). ANOVA statistically evaluates the significance of variance in each factor across different BPH infestation severity levels (Fisher, 1954). The integration of these three methods combines linear and nonlinear strengths to ensure a reliable and optimal selection outcome. Specifically, the evaluation scores from LDA, SHAP, and ANOVA were individually normalized and then averaged to form a joint determination indicator, which was used to assess the effectiveness of DSDI at various bands in differentiating BPH infestation severity.
2. The short-wavelength spectral region (399–750 nm) was divided into six subintervals, including violet (399–450 nm), blue (450–520 nm), green (520–580 nm), yellow-orange (580–630 nm), red (630–680 nm), and short-wavelength side of red-edge (680–750 nm). According to the peak positions of the joint assessment score, the most representative bands of DSDI were selected within the six short-wavelength spectral intervals and within the long-wavelength spectral region (750–1006 nm).

Based on the selected DSDI bands, the DSDI-SL were constructed via the normalized difference formula (Equation 3):


(3) $$\text{DSDI}-\text{SL}=\;\frac{{\text{D}}_{\text{long}-\text{wave}}-{\text{D}}_{\text{short}-\text{wave}}}{{\text{D}}_{\text{long}-\text{wave}}+{\text{D}}_{\text{short}-\text{wave}}}$$

where D_long-wave_ refers to the DSDI value corresponding to the selected band within the long-wavelength spectral region; D_short-wave_ refers to the DSDI value corresponding to the selected band within the short-wavelength spectral region. Thus, a set of candidates based on dual-temporal spectral differences of rice canopy was constructed.

#### 2.4.2 Feature selections

Hyperspectral remote sensing data provides an abundance of spectral information, thus introducing considerable redundancy. To enhance computational efficiency and mitigate the risk of overfitting problem, the candidate features were optimized and screened through a two-step process to ascertain the optimal feature combination for identifying BPH infestation:

1. Lasso regularization for feature reduction (Li et al., 2010). An L1 regularization term was incorporated into the objective function of logistic regression to shrink the coefficients of collinear features to zero. The optimal regularization parameter was identified via grid search, enabling the identification of the most informative features.
2. Recursive feature elimination (RFE) for feature optimization (Yan and Zhang, 2015): The fundamental concept of RFE involves iteratively modeling each input feature to assess its contribution and sequentially eliminating the least significant features based on their importance scores. The selection process was set to halt when no further significant improvement in model accuracy was observed, i.e., at a saturation point where the accuracy increase was less than 1%, thereby identifying the current set as the optimal feature combination.

### 2.5 BPH early infestation severity modeling and assessment

Three experimental scenarios were designed (**Table 2**). Specifically, a BPH early detection model was constructed using the proposed dual-temporal spectral difference-based features (i.e., DSRIs, DSDIs, and DSDI-SLs) in conjunction with pest severity labels derived from the averaged BPH population counts. For comparison, a mono-temporal spectral-based BPH early detection model was developed based on field records and hyperspectral imagery collected simultaneously.

**Table 2:** The designed experimental scenarios.

Scenario	Input features	Plot counts under different severity of BPH infestation		
		Mild	Moderate	Severe
Mono-0930	VIs calculated from hyperspectral images collected on Sep. 30, 2024	4 (1764 pixles)	45 (19845 pixles)	3 (1323 pixles)
Mono-1008	VIs calculated from hyperspectral images collected on Oct. 08, 2024	14 (6174 pixles)	33 (14553 pixles)	5 (2205 pixles)
Dual-DF	Proposed DSRIs, DSDIs, and DSDI-SLs	6 (2646 pixles)	42 (18522 pixles)	4 (1764 pixles)

Since the experiment was conducted during the early stage of BPH infestation, plots exhibiting moderate infestation severity predominated, leading to a significant class imbalance. Addressing this imbalance during the training phase is crucial to prevent the model from skewing towards classifying test samples into the majority class. There are two approaches to address this issue, including data augmentation for the minority class and downsampling for the majority class. Since pest infestation severity was assessed via a five-point sampling method in each plot, considerable pixel-level noise was already present under each label. Augmenting the minority class could potentially amplify this noise, adversely impacting model performance. Therefore, we adopted a ClusterCentroids downsampling approach for managing the majority class samples to balance the sample classes.

The samples were randomly split into independent training- and test-sets at a ratio of 7:3. The training-set was served for feature selection and the fine-tuning of model hyperparameters. XGBoost (extreme gradient boosting) was selected as the classifier for modeling BPH infestation severity. As an ensemble algorithm, XGBoost optimizes its objective function by sequentially incorporating weak learners (e.g., decision trees), iteratively correcting residual errors of previous models until reaching optimal performance. The grid search strategy is uniformly employed to determine the hyperparameters of XGBoost in each experimental scenario, including learning rate (LR), number of estimators (NE), maximum depth (MD), minimum child weight (MCW), and gamma (GAM). Subsequently, the model’s training performance was assessed using 10-fold cross-validation. The test-set was employed to evaluate the model’s generalization capabilities. Based on the confusion matrix from test-set results, the model’s ability to detect BPH infestation severity was evaluated using overall accuracy (OA), user accuracy (UA), and producer accuracy (PA). The aforementioned process was carried out using Python 3.9.19.

## 3 Results

### 3.1 Spectral characteristics of the rice canopy under BPH infestation

**Figure 3:**
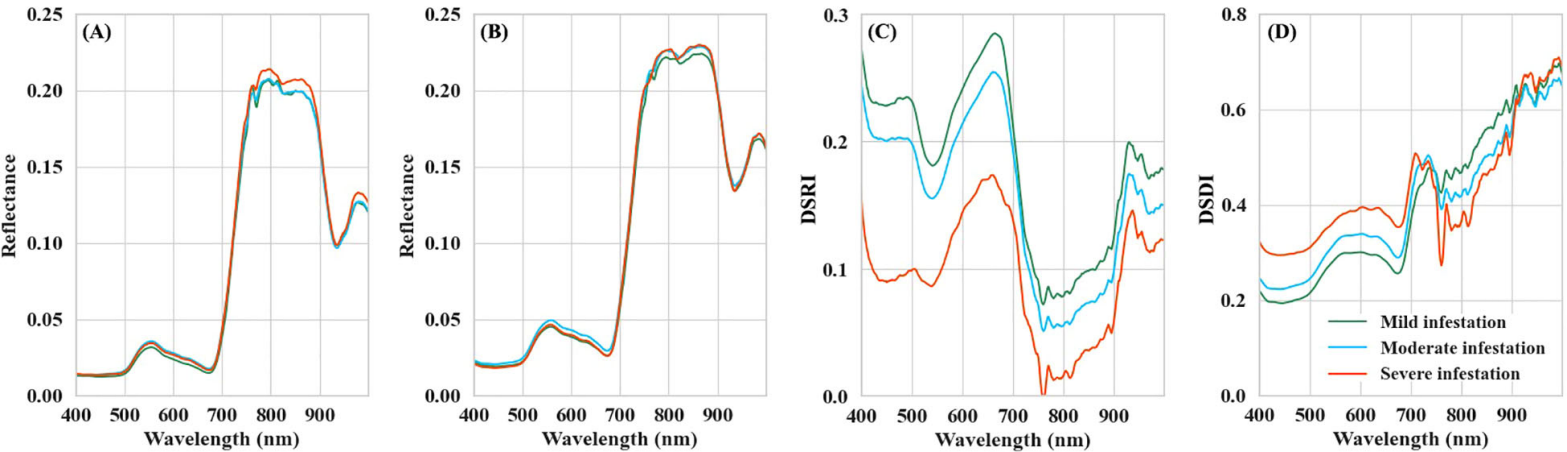
Optical characteristics of rice canopy under different BPH infestation severity: **(A)** reflectance data collected at T1; **(B)** reflectance data collected at T2; **(C)** the values of DSRIs; **(D)** the values of DSDIs.

The spectral differences of rice canopy across varying BPH infestation severities were first compared at T1 and T2 (**Figures 3A, B**). The spectrum curves of the three severity groups maintain typical peak-valley spectral features of vegetation. However, no consistent trend in reflectance was observed as infestation severity deteriorated. According to the spectral data collected at T1, the samples with mild infestation exhibited the lowest reflectance in the visible spectrum (399–680 nm), while the reflectance of moderate and severe infestation samples in this range was relatively similar. In the spectral region from 740 to 1006 nm, severe infestation samples showed the highest reflectance, whereas the mild and moderate infestation samples had comparable reflectance values. Regarding the data collected at T2, in the visible spectrum, the reflectance of the moderate infestation samples was the highest among the three groups, with the mild and severe infestation samples showing similar reflectance. In the NIR region, the reflectance of the mild infestation samples was generally lower than that of the moderate and severe infestation samples.

In contrast, the DSRIs and DSDIs values derived from the dual-temporal spectral differences of rice canopy were more sensitive to changes in BPH infestation severity (**Figures 3C, D**). For DSRIs, the mild infestation plots had the highest values, followed by the moderate infestation plots, and the values of severe infestation plots is the lowest. For DSDIs, the index values of infested rice increased with the severity of the infestation within the 399–750 nm spectral region. However, in the red-edge to NIR band range towards the long-wavelength direction (750–1006 nm), the DSDIs decreased as the infestation severity increased.

**Figure 4:**
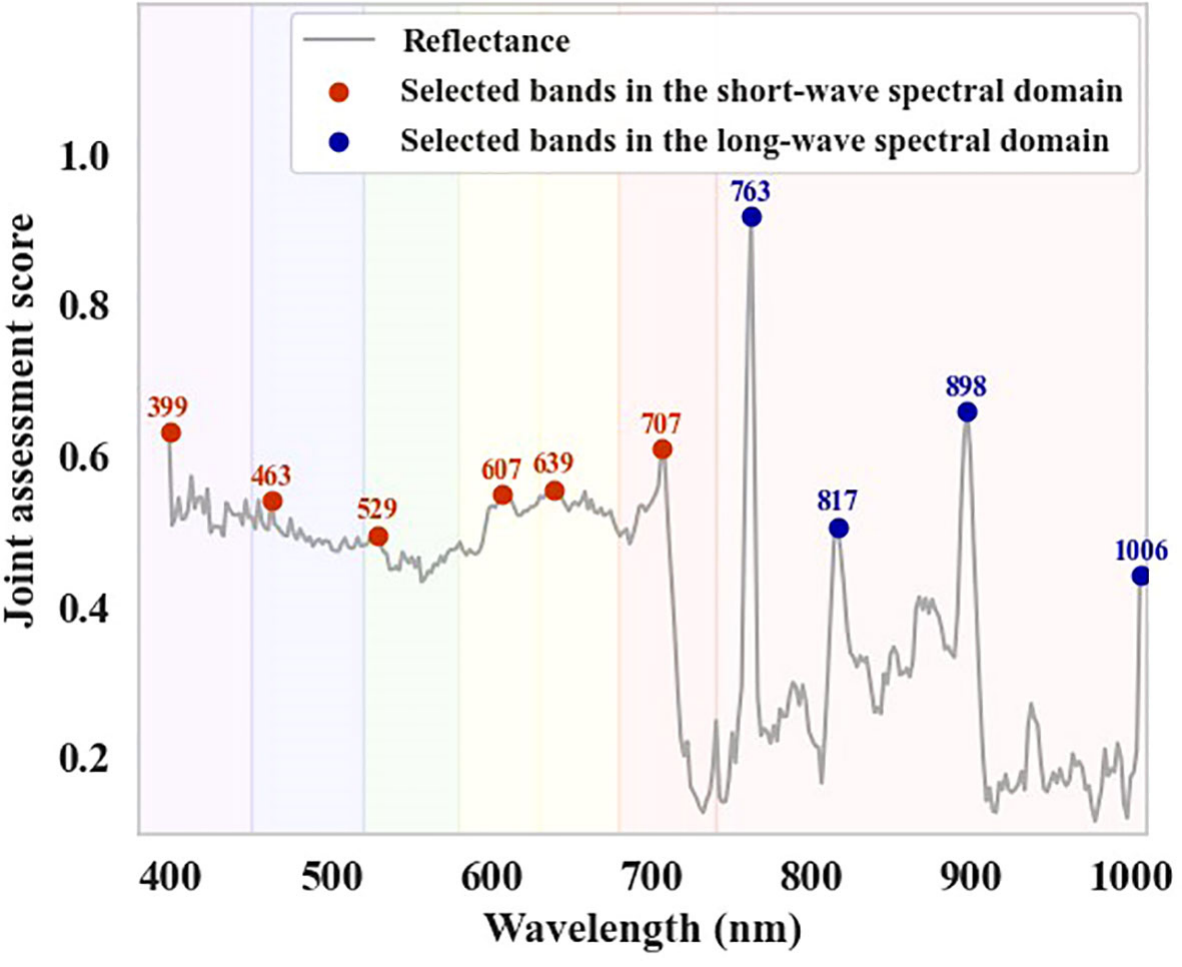
Selected bands through joint assessment for constructing DSDI-SL.

**Figure 5:**
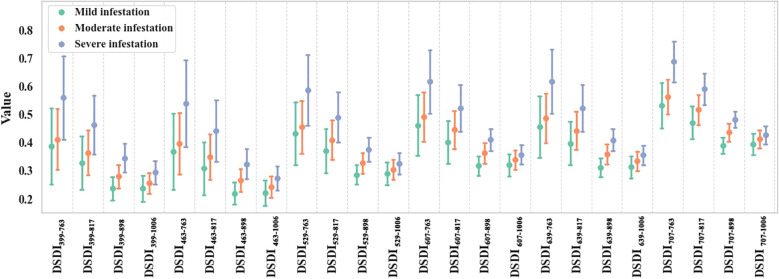
Variations of DSDI-SLs under different severities of BPH infestation.

Based on the peak positions of the joint assessment scores, the representative bands of DSDI for constructing the DSDI-SL were selected (**Figure 4**). For the six subintervals in the short-wavelength spectral region, the selected DSDI were located at 399 nm, 463 nm, 529 nm, 607 nm, 639 nm, and 707 nm. In the long-wavelength spectral range, four representative DSDIs were selected, corresponding to the wavelengths of 763 nm, 817 nm, 898 nm, and 1006 nm. Using the formula for constructing DSDI-SL, a total of 24 candidates were developed. As shown in **Figure 5**, the values of these indices all exhibited an increasing trend with the deterioration of infestation severity.

### 3.2 Feature selection results

**Figure 6:**
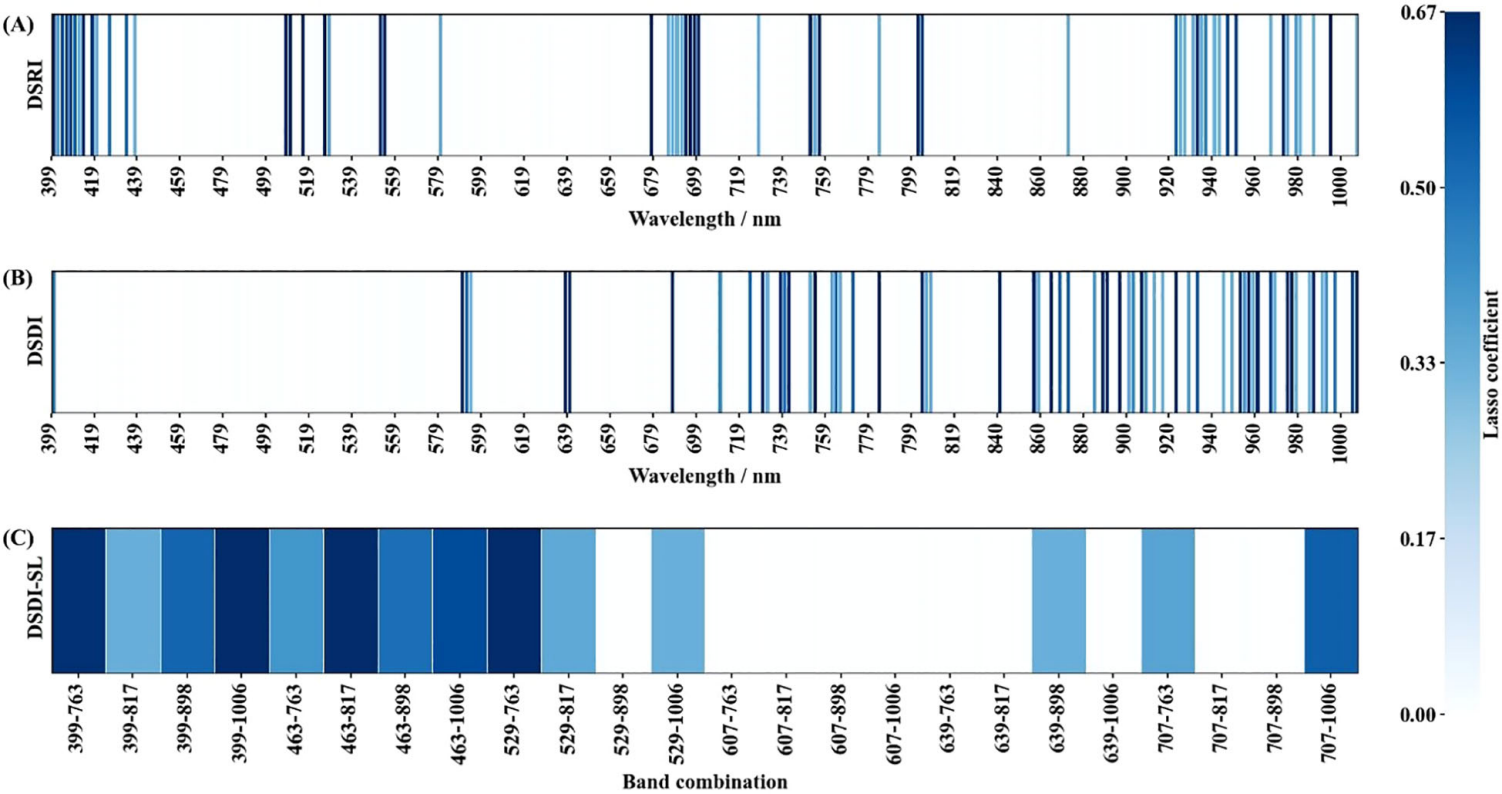
Results of collinearity removal for **(A)** DSRI, **(B)** DSDI, and **(C)** DSDI-SL using Lasso.

A total of 632 dual-temporal spectral difference-based candidates were constructed (DSRIs: 304, DSDIs: 304, DSDI-SLs: 24). Among them, 499 candidates exhibiting high collinearity, i.e., with a Lasso coefficient equal to 0, were eliminated (**Figure 6**). Following regularization, the selected DSRIs are mainly distributed in the violet, blue, red-edge, and long-wavelength side of the NIR region. Representative DSDIs are mostly located in the yellow-orange, red, and the red-edge to NIR regions. For the selected DSDI-SLs, the construction formula that incorporate DSDIs from the violet, blue, and green bands exhibited the highest selection rate.

**Figure 7:**
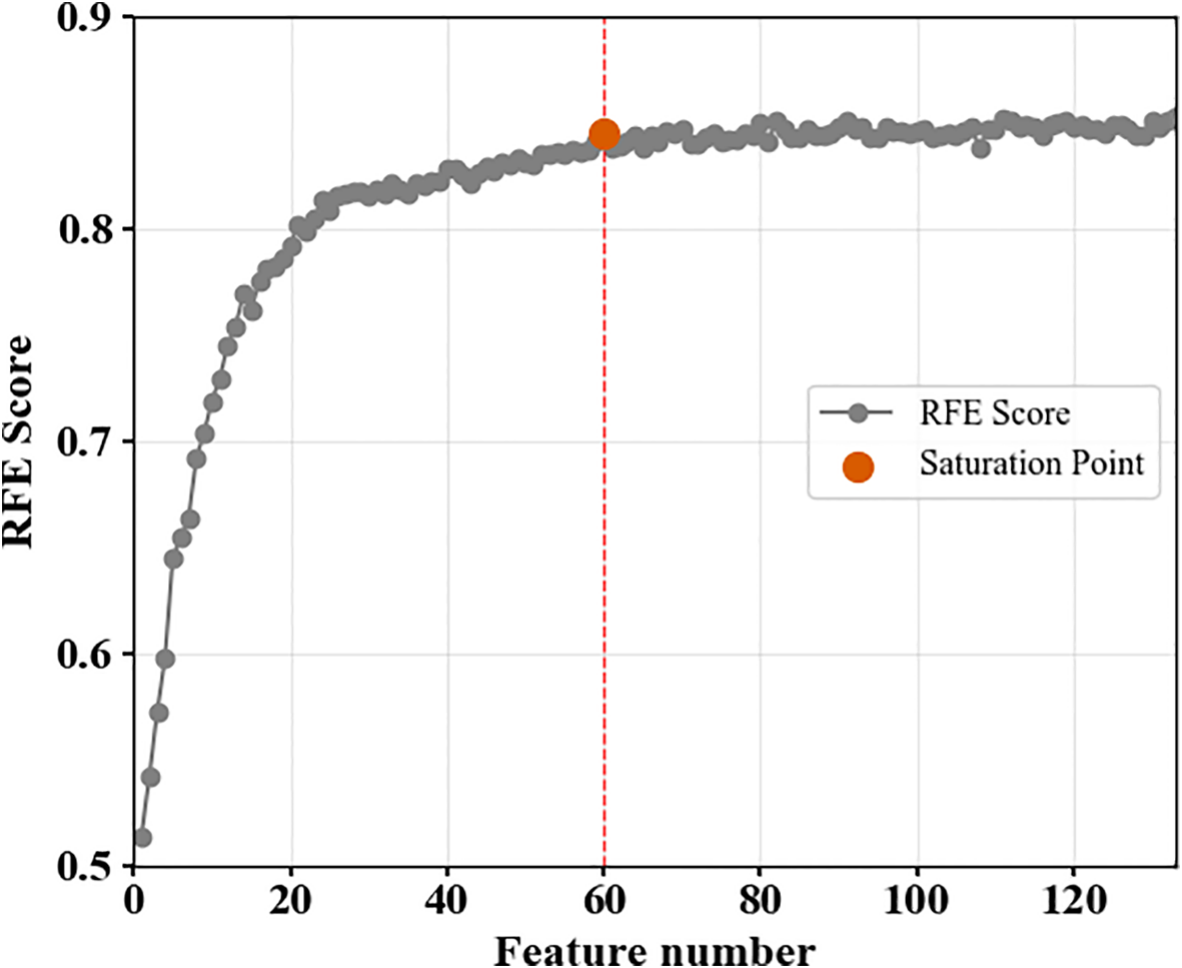
The selection results of RFE.

The optimal features combination for BPH identification was further determined through RFE. According to the iterative results, the model’s accuracy saturated when the number of selected features exceeded 60 (**Figure 7**). The selected DSRIs constituted one-third of the total features, with over 70% found in the violet, blue, and green spectral regions. The remaining four DSRI features are distributed across the red-edge and NIR bands. The selected DSDIs accounted for more than half of all features, predominantly in the red-edge and NIR regions, with counts of 11 and 23, respectively. Four DSDI-SL were selected, three of which were constructed using DSDIs from the near-infrared and violet-blue regions, and the other one was constructed with two DSDI from the red-edge region (**Table 3**).

**Table 3:** The 60 selected dual-temporal spectral differences-based features.

Features type	Selected features
DSRI	DSRI_399_, DSRI_401_, DSRI_403_, DSRI_405_, DSRI_407_, DSRI_409_, DSRI_411_, DSRI_413_, DSRI_417_, DSRI_425_, DSRI_433_, DSRI_437_, DSRI_525_, DSRI_553_, DSRI_579_, DSRI_697_, DSRI_699_, DSRI_727_, DSRI_753_, DSRI_803_, DSRI_932_
DSDI	DSDI_399_, DSDI_709_, DSDI_729_, DSDI_739_, DSDI_741_, DSDI_751_, DSDI_753_, DSDI_761_, DSDI_763_, DSDI_765_, DSDI_771_, DSDI_783_, DSDI_803_, DSDI_807_, DSDI_856_, DSDI_896_, DSDI_902_, DSDI_922_, DSDI_928_, DSDI_932_, DSDI_952_, DSDI_954_, DSDI_958_, DSDI_960_, DSDI_968_, DSDI_974_, DSDI_976_, DSDI_978_, DSDI_984_, DSDI_986_, DSDI_990_, DSDI_992_, DSDI_996_, DSDI_1004_, DSDI_1006_
DSDI-SL	DSDI-SL_399-817_, DSDI-SL_399-1006_, DSDI-SL_465-1006_, DSDI-SL_707-763_

### 3.3 BPH infestation severity models and accuracies

The model hyperparameters and OA values for the three scenarios were determined during the training process (**Table 4**). The OA values for both the training- and test-sets across the three scenarios were similar, indicating that the model did not overfit. From the training results, the Dual-DF scenario achieved the highest accuracy, with OA values surpassing 85% for both sets. The Mono-0930 scenario followed, with an OA approaching 80%, whereas the Mono-1010 scenario recorded an OA of about 75%.

**Table 4:** The calibrated hyper-parameters and accuracy of each scenario.

Scenario	Hyper-parameters					OA (%)	
	LR	NE	MD	MCW	GAM	Training-set	Test-set
Mono-0930	0.35	200	6	1	0	79.53 ± 0.38	79.89
Mono-1008	0.3	370	6	2	0	74.45 ± 0.48	75.91
Dual-DF	**0.28**	**340**	**6**	**1**	**0**	**85.15 ± 0.61**	**85.10**

Bold values represent the hyperparameters and identification accuracy of the two-phase model constructed in this study.

**Figure 8:**
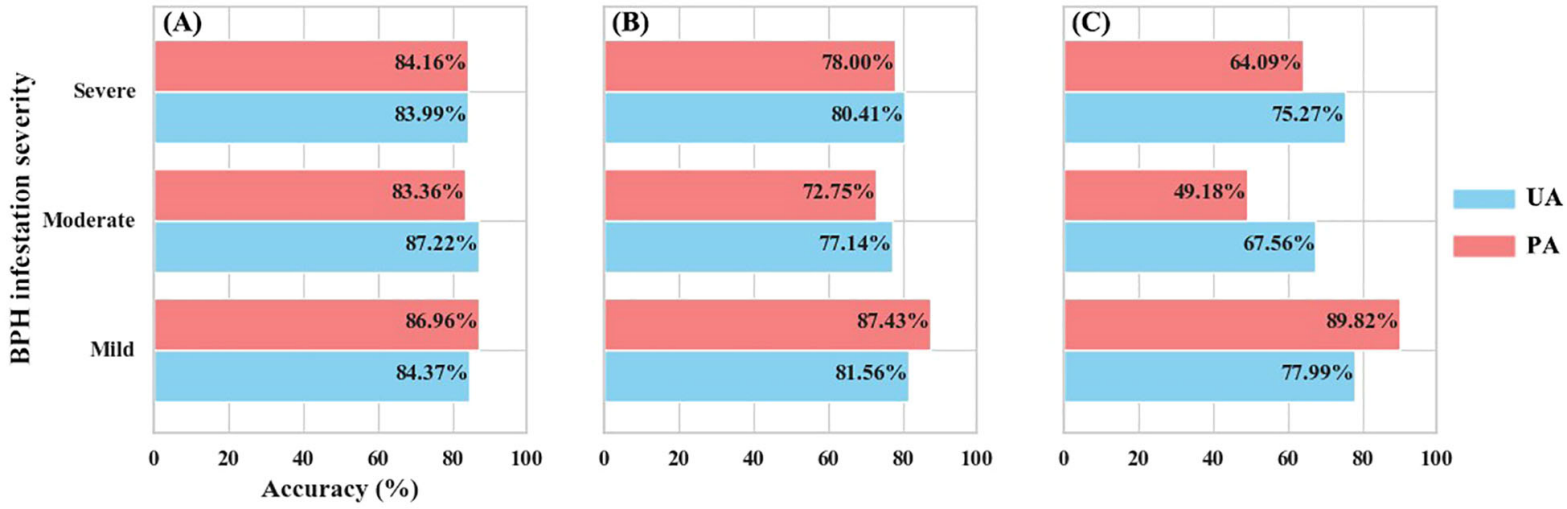
User’s (UA) and producer’s (PA) accuracy of the test-set: **(A)** Dual-DF; **(B)** Mono-0930; **(C)** Mono-1008.

The detection accuracy of both dual- and mono-temporal scenarios for different BPH infestation severity was further assessed using the test-set results (**Figure 8**). All three scenarios performed well in identifying samples with mild infestation, with both PA and UA exceeding 80% for the Dual-DF and Mono-0930 scenarios. The Mono-1008 scenario achieved a PA close to 90% for mild infestation but had a UA below 78%. For samples with moderate and severe infestations, the performance of the mono-temporal feature models was less satisfactory. The accuracy for moderate infestation samples was below 80% for Mono-0930 and below 70% for Mono-1008. The PA of Mono-1008 dropped below 50%. Although the mono-temporal feature models performed slightly better when identifying severe infestation samples, both their PA and UA remained consistently lower than those achieved by the Dual-DF scenario.

**Figure 9:**
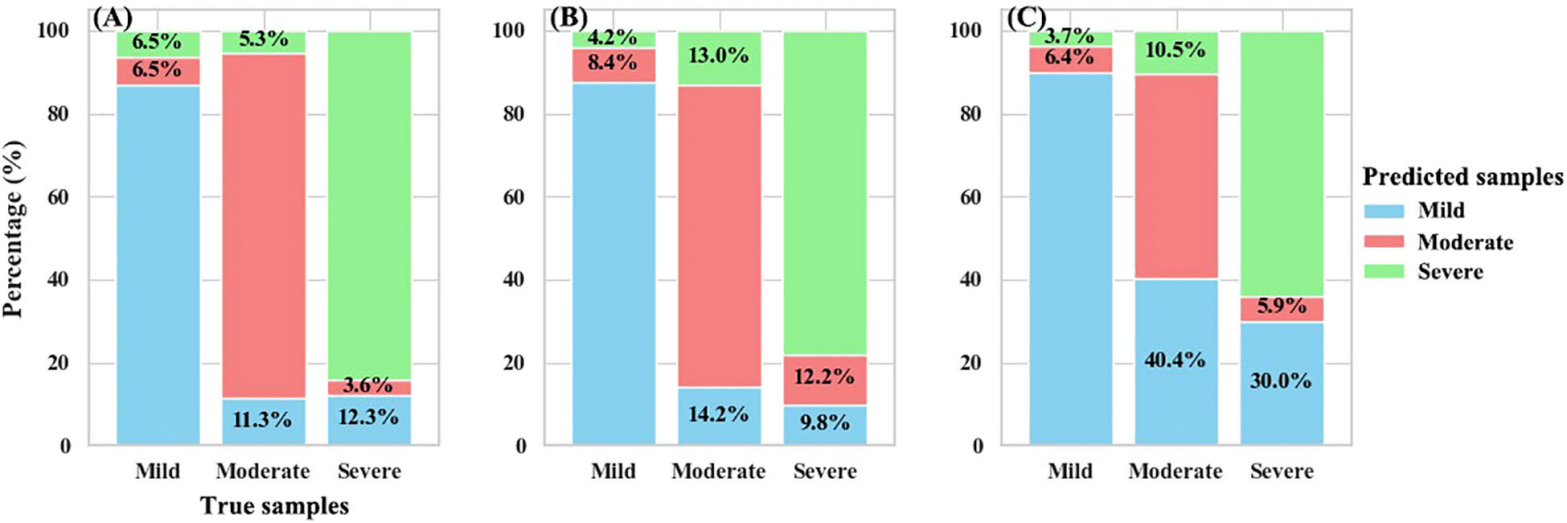
Misclassification rate of scenarios: **(A)** Dual-DF; **(B)** Mono-0930; **(C)** Mono-1008.

We further examined the misclassification rates across different BPH severities for the three scenarios (**Figure 9**). Overall, all three scenarios exhibited relatively low misclassification rates for samples with mild infestation. The primary sources of model error arose from the identification results of the moderate and severe infestation severity groups. For Dual-DF, there was a tendency to misclassify moderate and severe infestation samples as mild. For Mono-0930, the highest misclassification rate was for moderate infestation samples, with 14.2% incorrectly identified as mild and another 13% misclassified as severe. For Mono-1008, its performance on moderate and severe samples was suboptimal, with the model misclassifying 40% of moderate infestation samples and 30% of severe infestation samples as mild.

## 4 Discussion

### 4.1 Uncertainty in mono-temporal spectral features for monitoring early BPH infestation

Capturing the degradation signals of host physiological traits and canopy morphology is fundamental for remote sensing monitoring of insect disturbances. The prevailing consensus suggests that spectral information spanning from the visible to the red-edge spectrum can be utilized to detect alterations in host pigment content, whereas signals in the NIR and shortwave infrared (SWIR) regions are particularly responsive to changes in host structure and moisture levels (Zhang et al., 2019; Zheng et al., 2023). By selecting specific spectral features aligned with the feeding behaviors of insects, e.g., folivores, xylophages, and mucivores, it becomes possible to quickly diagnose both the location of infestation and the severity of stress caused by the target pest. Some researchers have conducted controlled experiments with pest populations to elucidate the physiological responses of rice to BPH infestation (Chen and Liu, 2023; Zhao et al., 2023).

Compared to the stable conditions of controlled indoor experiments, pest monitoring studies in paddy fields are subject to numerous interfering factors. The population density of BPH per unit area is profoundly influenced by multiple factors, e.g., field migration and reproductive behavior of BPH (Mochizuki et al., 2024). In mono-temporal scenarios, the rice spectra in areas with high BPH population density may not display marked changes. This is because BPH abundance may changes sharply within a short period, while the rice canopy has not yet exhibited notable distortion (**Table 5**). Meanwhile, in manually sown paddy fields, variations in planting density across locations may result in differences in canopy spectral characteristics, as remote sensing pixels in sparsely planted areas are more likely to include background signals from soil or water. Consequently, a BPH identification model derived from mono-temporal spectral features introduces considerable uncertainty.

**Table 5:** A portion of the recorded BPH population counts.

Plot ID	BPH population (individuals per clump)	
	T1	T2
1	6	3.8
2	7.6	7
3	9.2	7.2
4	10.2	8.8
5	10.4	7.4
…	…	…
26	7	24.8
27	7.2	13.2
28	6.8	12
29	7	4.6
30	11.4	9.2
31	9.2	14.2
…	…	…
48	5.8	5.8
49	6	8.8
50	5.8	4.6
51	5.6	4.4
52	5.8	4.6

According to the modeling results using mono-temporal spectral features, the identification accuracy of model is relatively higher when the investigation date of input features is earlier. This could be attributed to the fact that the earlier the investigation time, the smaller the change in pest population density per unit area. As BPH infestation progresses dynamically, the likelihood of changes in pest population density within each plot increases. The cumulative pest stress on rice exhibits greater spatial variability, which reduces the pest identification accuracy of the mono-temporal spectral-based model.

### 4.2 Detectability of dual-temporal spectral features in early BPH infestation monitoring

From the perspective of agricultural production, the timely identification of paddy areas under mild stress (i.e., low population density, in the initial stage of infestation) is a prerequisite for the precise control of BPH damage. However, given that BPH do not directly attack the leaves, the degree of spectral changes in the rice canopy is marginal during the early stages of infestation, i.e., the phenotype of rice shows no visible signs of deterioration, and its spectrum retains the typical characteristics of healthy green vegetation. Interestingly, in the NIR region, which is typically an important indicator of vegetation health, samples with severe infestation even exhibited slightly higher reflectance than those with milder infestations. Evidently, this result is inconsistent with previous research (Liao et al., 2024; Yang et al., 2024).

As mentioned in Section 4.1, mono-temporal spectral features are susceptible to multiple sources of field noise when monitoring BPH infestation. To address this issue, we propose a novel method for monitoring BPH early infestation that uses dual-temporal spectral difference features to mitigate such interference. Specifically, we focused on the following two aspects during construction process: (1) Considering the uncertainty of using single-time BPH population data as training labels, we used the average of BPH population counts from two sampling dates instead. Since the interval between the two sampling dates was relatively short (8 days), this approach provides a more reliable assessment of BPH damage severity during this period and reduces the randomness inherent in single-time counts. (2) A set of feature indices (i.e., DSRIs, DSDIs, DSDI-SLs) was proposed to detect early BPH infestation based on dual-temporal spectral differences in rice canopies. By treating inherent variations (e.g., rice growth, background flooding, planting density difference) as a baseline, these indices effectively highlight spectral anomalies induced by BPH infestation, thereby reducing the interference of non-BPH factors on identification results.

According to the “Rules of investigation and forecast for the rice planthopper (*Nilaparvata lugens* Stål and *Sogatalla furcifera* Horváth) (GB/T 15794-2009)” and the “Technical regulations for comprehensive control of major pests affecting high quality rice in Guangdong (DB44/T 2212-2019)”, control measures should be implemented when the BPH counts per rice clump exceed 10. Since the training labels used in the proposed model were derived from the average of two field surveys, an extreme case may arise (i.e., when the BPH population is 0 in one survey but exceeds 10 individuals per rice clump in another). Therefore, in practical applications, control measures are recommended for areas identified by the proposed model to have moderate-to-severe BPH damage. The experimental results demonstrated that the proposed model achieved an accuracy of at least 83% for samples with moderate and severe damage, indicating its potential for practical application.

### 4.3 Limitations and further studies

**Figure 10:**
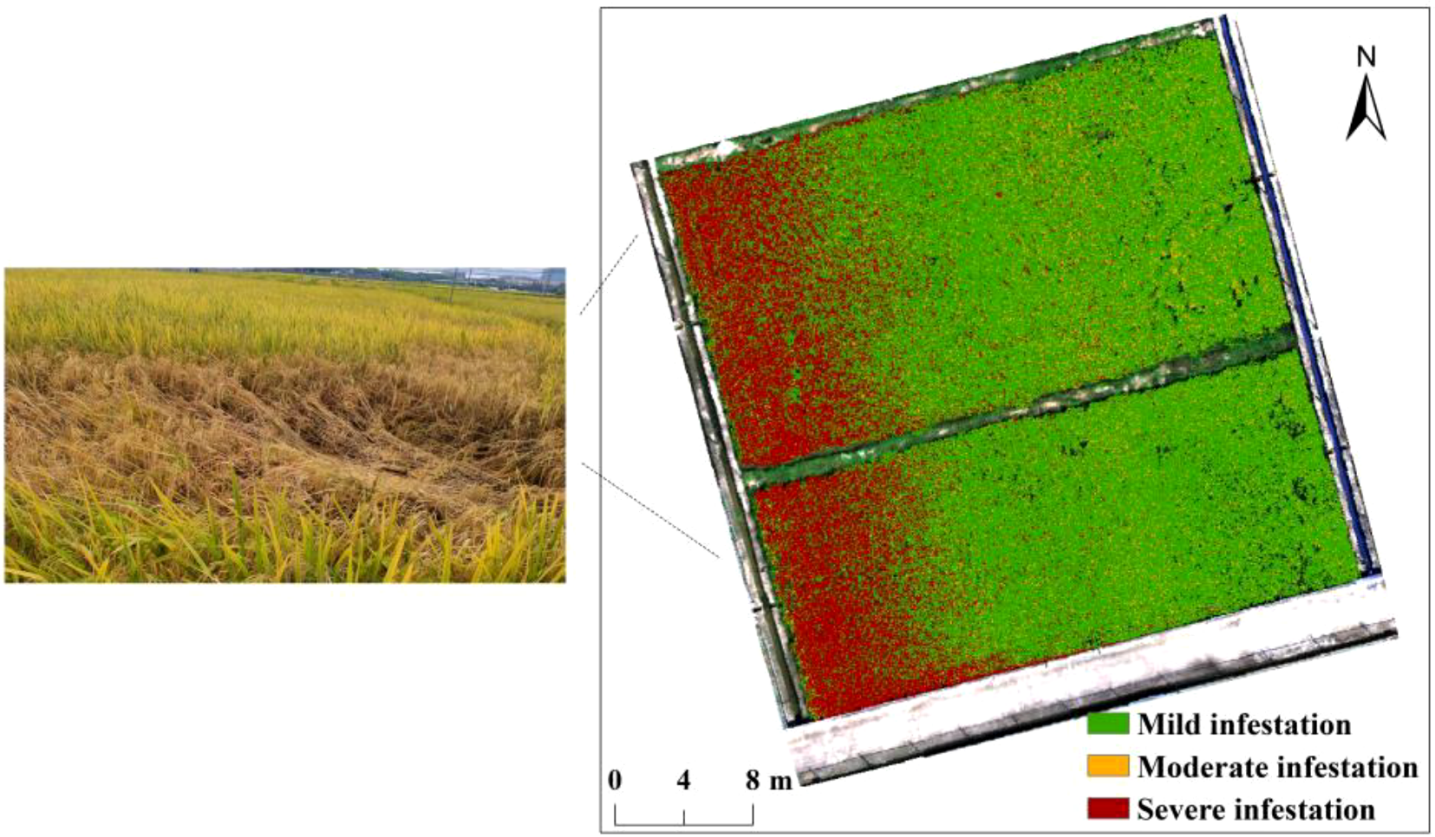
The spatial distribution of BPH infestation in paddy field.

Overall, the proposed BPH early detection model has achieved promising results. However, it still has certain limitations that merit consideration in further studies:

1. In this study, the proposed dual-temporal spectral indices (i.e., DSRI, DSDI, and DSDI-SL) were confirmed to effectively monitor early BPH damage, but uncertainties regarding the occurrence timing remain due to the migratory behavior of BPH. Since the nutrient content of rice varies across different growth stages, its physiological responses to BPH infestation differ accordingly (Zhao et al., 2023; Yang et al., 2024). Therefore, the detectability of DSRI, DSDI, and DSDI-SL for early BPH infestation across different rice growth stages will be further evaluated, aiming to enhance their practical applicability.
2. According to the distribution map of early BPH infestation generated by the proposed model (**Figure 10**), the areas severely infested by BPH were primarily located on the southwestern side of paddy field. The rice lodging event caused by BPH was observed about 25 days later in this area, thereby demonstrating the effectiveness of the proposed model. Moreover, this finding also reflects the influence of the rice sub-canopy environment on BPH aggregation. The terrain in the southwestern part of the paddy field is lower, which results in a wetter and cooler sub-canopy environment, potentially facilitating the aggregation of BPH (Mochizuki et al., 2024). Therefore, auxiliary habitat features (e.g., soil moisture, temperature, and elevation) will be incorporated to further explore how habitat heterogeneity impacts the population density of BPH within the paddy field.
3. The sap-sucking feeding behavior of BPH primarily affects the nutrient transport system of rice, the variation in water content of infested rice is theoretically a critical indicator for evaluating BPH infestation severity (Chen and Liu, 2023; Xiong et al., 2024; Yue et al., 2024). However, the spectral range of the hyperspectral imaging system used in this study was limited to 399–1006 nm, we were unable to evaluate the effectiveness of water content in monitoring BPH infestation due to the lack of SWIR information. To gain a more comprehensive understanding of the capability of remote sensing for early BPH infestation detection, the potential contribution of high-resolution SWIR data will be further investigated in future studies.

## 5 Conclusions

This study successfully identified early BPH infestation using UAV hyperspectral observation data. Considering the dynamic spatial distribution of BPH and the reflectance changes in infested rice, three novel dual-temporal spectral indices, i.e., DSRI, DSDI, and DSDI-SL, were proposed. By integrating Lasso regularization and RFE (for optimal feature selection) with XGBoost (for classifying BPH infestation severity), a model for BPH early detection was developed. The model achieved an OA of over 85%. It’s PA and UA for samples across varying BPH severity at least 83%, notably outperforming models derived from mono-temporal spectral-based features. In contrast, mono-temporal spectral-based model is susceptible to dynamic changes in BPH population density per unit area and other inherent factors (e.g., rice growth, background flooding, differences in planting density), leading to considerable uncertainty in the detection outcomes.

## Data Availability

The original contributions presented in the study are included in the article/supplementary material. Further inquiries can be directed to the corresponding author.
